# Audience Response System (ARS) Use in the SCORE (Surgical Council on Resident Education) Surgery Training Curriculum: A Mixed Methodology Study

**DOI:** 10.7759/cureus.44721

**Published:** 2023-09-05

**Authors:** Faiz Tuma, Anastasiya Shchatsko, Mohamed Kamel, Joseph Vyskocil, John Blebea

**Affiliations:** 1 Surgery, Central Michigan University College of Medicine, Saginaw, USA

**Keywords:** educational technology, interactive, education, training, residency, audience response system

## Abstract

Background

Audience Response Systems (ARS) could help overcome the limitations of traditional lectures by providing interactivity, engagement, and assessment. The perception of ARS use in surgical education is not well documented.

Objective

Examine the use of an ARS in teaching *This Week In SCORE* (Surgical Council on Resident Education)sessions to general surgery residents and medical students.

Methods

ARS was used at weekly SCORE question sessions in a new general surgery residency program by four residents, 97 medical students, and 20 faculty. The study employed a mixed quantitative and qualitative method: two separate 10-question surveys for faculty and trainees (49% response rate) and a focus group discussion that included one faculty member, two residents, and two students.

Results

In 85 (85%) responses, the faculty favored the use of ARS in SCORE. Among the total of 510 responses from 51 residents and students, 57% agreed with the favorable use of ARS, while 28% were neutral and, in 14% of cases, negative. A greater proportion of faculty and learners preferred ARS over traditional lectures. The focus group content analysis showed a positive effect and preference from learners and faculty. Engagement, thinking stimulation, and group participation were the most common positive comments. No significant negative influence on ARS use was reported.

Conclusions

The use of an ARS in *This Week In SCORE* ​​​sessions were preferred by most of the faculty and a majority of learners. The benefits are ease of use and stimulation of discussion. ARS has the potential for more widespread utilization in additional educational settings.

## Introduction

Postgraduate education is challenging and requires utilizing a highly collaborative and interactive educational environment. In the process of training, especially during didactic conferences, it is essential to deliver information and evaluate residents’ and students` knowledge in the most efficient manner, especially for surgical training programs that have limited time for classroom education. Of all medical education teaching methods, the lecture is by far the most common, allowing for efficient knowledge transfer to large groups of trainees [1]. At the cost of higher learner passivity [2], large-group lectures are an efficient teaching method [3] but are subject to inherent limitations. They frequently lack sufficient interactivity and learners’ engagement, as well as limited capabilities for knowledge assessment. Group discussions and problem solving provide greater interactivity but do not give an equal voice to all trainees.

Educational technology can potentially overcome these limitations by providing for large-group information transfer along with full learner interactivity and individual engagement and assessment. An Audience Response System (ARS) is a technological tool that can encourage participation in a non-threatening manner, instantly collect learners’ responses, and display them in a way that is visually engaging and easy to comprehend [4]. ARS has been used in medical didactic sessions and morbidity and mortality conferences [5-7], with evidence that it improves knowledge acquisition and retention [8]. However, there are limited studies on the use of ARS to enhance interactive surgical education or its use in association with the Surgical Council on Residents Education (SCORE; www.surgicalcore.org) curriculum for surgical residents and medical students. Agreeing on the importance of learner engagement [9], we hypothesized that surgical education will benefit from the utilization of a more interactive environment provided by ARS and will be perceived well.

This study was designed to evaluate the perception of ARS use in teaching SCORE weekly sessions by assessing learners’ and faculty experiences through a mixed quantitative and qualitative methodology. These results may encourage other surgical and non-surgical/residency programs to implement ARS for residents and medical students in association with both the SCORE curriculum and other teaching conferences.

## Materials and methods

The study was conducted within a department of surgery to describe and evaluate experiences with interactive education. *This Week In SCORE* sessions were held once a week for an hour as part of academic education sessions for first-year surgical residents and 3rd/4th-year medical students participating in their surgical clinical rotation. The sessions involved a faculty moderator leading a discussion utilizing the 10 SCORE questions covering the curriculum and reading material of that week. Residents and students participated in answering the SCORE questions anonymously. The ARS technology (Turning Point; Turning Technologies, Youngstown, OH) employed a small hand-held response device that allowed individual residents and students to answer the SCORE questions with immediate group answers displayed on a large screen. The distribution of answers provided the faculty moderator with direction for further group discussion on the topic.

At the end of the academic year, all faculty, residents of the surgery program, and medical students who had been on the surgical rotation were invited to participate in a survey questionnaire prepared for this study. Two surveys, one for faculty (Appendix) and another for residents and students (Appendix B), were designed for quantitative analysis. Each questionnaire consisted of 10 multiple-choice questions designed on a 5-level Likert scale to assess the participants’ perceptions of the educational value, feasibility, practicality, and overall experience of utilizing ARS. Chi-square and Fisher’s exact tests were used to compare faculty and learners’ responses, with a p < 0.05 considered statistically significant. The Central Michigan University Institutional Review Board reviewed the study proposal and determined the study to be exempt from individual informed consent.

Focus group discussion

A focus group discussion (FGD) was conducted for qualitative analysis of the perceptions of the participants concerning the use of ARS. This method is useful in participatory research [10] to provide further depth and detail to quantitative data [11]. Five participants (two residents, two students, and one faculty member) who agreed to participate in the FGD were selected. The in-person discussion focused on their experience using the ARS and was moderated by the primary investigator (FT). The opening question, "How was your experience using the ARS?" was used to start the discussion. Free participation was allowed with appropriate moderation to keep the discussion focused on their experience using the ARS. A neutral stand by the moderator was maintained while participation time and speaking opportunities were allowed equally to all participants. All responses and observations from the session were recorded, and the content was later analyzed [12]. As described by Krippendorff [13], content analysis was performed by classifying words from the discussion into content categories and thematic units [14].

## Results

There was an overall 49% response rate to the surveys. Twenty faculty used the ARS system in their SCORE teaching sessions, and 10 (50%) responded to the survey. All four of our new surgical residents used the system, and all 100% responded. Of the 97 medical students who participated in the teaching sessions, 47 (48%) completed the surveys.

Of the 100 faculty question responses, 85 (85%) either agreed or strongly agreed on the favorable use of ARS in SCORE, while 10 (10%) were neutral and five (5%) disagreed (Table 1). The most preferred features of the ARS system for faculty were as follows: engaging learners, efficient use of educational time, and encouraging the use of technology in education. In addition, 60% of the participating faculty either agreed or strongly agreed that it was educationally better than traditional lectures.

**Table 1:** Using ARS and SCORE – faculty survey

	Strongly agree	Agree	Neutral	Disagree	Strongly disagree
Q1	Needed an acceptable amount of preparation time and effort				
	4 (40%)	5 (50%)	0	1 (10%)	0
Q2	Helped learners’ engagement and participation				
	5 (50%)	5 (50%)	0	0	0
Q3	Helped knowing learners’ level and facilitated building on their knowledge				
	3 (30%)	4 (40%)	3 (30%)	0	0
Q4	Facilitated learning relevant to clinical activities				
	2 (20%)	6 (60%)	2 (20%)	0	0
Q5	Efficient use of learning time				
	5 (50%)	5 (50%)	0	0	0
Q6	Educationally better than traditional lectures				
	3 (30%)	3 (30%)	3 (30%)	1 (10%)	0
Q7	Appropriate way to teach the topic				
	2 (20%)	7 (70%)	0	1 (10%)	0
Q8	Encouraged using technology in training				
	5 (50%)	5 (50%)	0	0	0
Q9	Facilitated learning concepts and principles rather than factual information				
	3 (30%)	4 (40%)	1 (10%)	2 (20%)	0
Q10	Good quality educational activity				
	4 (40%)	5 (50%)	1 (10%)	0	0

There were no statistically significant differences between resident and student responses (P =0.62 in the Fisher’s exact test). Therefore, they were combined for comparison purposes. Among the total of 510 question responses from 51 learners (residents and students), the majority appreciated the ease of use, engaging effect, and stimulation of discussions from the use of ARS during the SCORE session. The majority of the responses, 293 (57%), either agreed or strongly agreed with the favorable use of ARS, 145 (28%) were neutral, and 72 (14%) disagreed or strongly disagreed (Table 2).

**Table 2:** Using ARS and SCORE – trainee survey

	Strongly agree	Agree	Neutral	Disagree	Strongly disagree
Q1	Easy and practical				
	9 (18%)	27 (53%)	12 (24%)	3 (6%)	0
Q2	Motivational and engaging				
	7 (14%)	26 (51%)	11 (22%)	5 (10%)	2 (4%)
Q3	Stimulated study and learning				
	6 (12%)	25 (49%)	12 (24%)	5 (10%)	3 (6%)
Q4	Helped better understand and apply knowledge				
	6 (12%)	22 (43%)	16 (31%)	4 (8%)	3 (6%)
Q5	Encouraged using technology in learning				
	6 (12%)	16 (31%)	20 (39%)	6 (12%)	3 (6%)
Q6	Encouraged discussion with colleagues and faculty				
	7 (14%)	25 (49%)	14 (28%)	2 (4%)	3 (6%)
Q7	Improved quality of learning and focusing on principles and concepts				
	8 (16%)	25 (49%)	13 (26%)	3 (6%)	2 (4%)
Q8	Efficient use of learning time				
	9 (18%)	21 (41%)	11 (22%)	5 (10%)	5 (10%)
Q9	Inspired learning and study topics further				
	5 (10%)	15 (29%)	22 (43%)	7 (14%)	2 (4%)
Q10	Prefer compared to traditional lecture or discussion				
	12 (24%)	16 (31%)	14 (28%)	5 (10%)	4 (8%)

Three questions were formulated similarly for the faculty and the trainees (Table 3). A majority of both groups felt that the addition of ARS was preferred and better than traditional lectures. All the faculty felt that utilizing ARS was a more efficient use of learning time and encouraged them to use more technology in teaching, but significantly fewer learners shared this perspective. 

**Table 3:** Comparison of responses between faculty and trainees

Questions (Faculty/Learners)		Agree	Neutral/Disagree	P-value
Q5/Q8	Efficient use of learning time			
	Faculty	10 (100%)	0	0.001
	Trainees	22 (45%)	29 (55%)	
Q6/Q10	Better than traditional lecture/Prefer ARS			
	Faculty	6 (60%)	4 (40%)	0.76
	Trainees	28 (55%)	23 (45%)	
Q8/Q5	Encouraged use of technology			
	Faculty	10 (100%)	0	0.01
	Trainees	30 (59%)	21 (41%)	

FGD content analysis

Content analysis was used to analyze the result of the FGD. Thematic units, which include more global interpretative or explanatory sets of statements, were used for content analysis. Recurring systems of beliefs or explanations represent thematic units. FGD content analysis showed that the learners and faculty instructors reported positive influence and preference in most of their comments. After coding and reviewing the FGD content, the following categorization appeared distinct: advantages and disadvantages. Within each category, subcategories were identified, and frequencies were noted (Table 4). 

**Table 4:** Focus group discussion – content categories

	Advantage	Frequency	Disadvantage	Frequency
1	Improving focus	3	Time-consuming	3
2	Thinking stimulation	6	Repetitive	1
3	Group participation	4		
4	Enjoyable	2		
5	Engagement	5		
	TOTAL:	20	TOTAL:	4

The total frequency in all categories was 24. Therefore, the favorable advantage frequency percentage was 83% (20/24), while the unfavorable disadvantage frequency was 17% (4/24). Engagement, thinking stimulation, and group participation were the most common positive comments. The disadvantages or issues that were identified with ARS use were time consumption and repetitiveness.

## Discussion

This study was designed and conducted to collect quantitative data using a survey questionnaire, followed by qualitative data collection through FGD, representing an explanatory sequential design method [11]. Such a mixed-methods technique provides greater detail and depth to quantitative data [15]. FGD methods have been used in academic settings to assess participants’ perceptions [10]. It provides an opportunity for individuals to express their opinions freely and discuss their impressions among themselves. This facilitates exploring and understanding, to a greater degree, the results from surveys, which otherwise would be difficult to explain from survey data alone [12].

Our study was conducted to evaluate faculty, residents, and medical students’ perceptions of ARS use as an educational technology tool in *This Week In SCORE* sessions. Study results revealed that both faculty and learners reported overall positive effects of ARS utilized during SCORE didactic sessions. For the faculty, 90% felt that incorporating ARS into their teaching sessions did not require an inordinate amount of preparation time or effort. They perceived it to be an appropriate and helpful way to teach, was efficient, facilitated the learning of concepts and principles, encouraged learners’ participation, and provided faculty with a better way to assess the learners level of knowledge. The large majority felt that it was a good educational activity and better than traditional lectures.

Learners-residents and students-also had an overall positive experience with ARS, and 55% preferred it as compared to a traditional lecture or discussion. For them, it was easy and practical to use, engaged their participation, helped their understanding of the topic, and encouraged further discussion. This is consistent with a large body of research suggesting that residents and students respond positively to the use of ARS in medical education [7,16]. Our modification was to incorporate ARS in the context of *This Week In SCORE* and the associated multiple-choice questions (MCQ) component of the SCORE curriculum. 

The benefits of ARS are plentiful and well documented in the literature. Overall, ARS improves teaching efficiency by targeting and devoting more attention to the most problematic topics. The revealed wrong answers become the focus of the discussion and attention, while questions answered correctly by all or most of the participants will only be briefly discussed. The immediate polling provided by ARS identifies gaps in the learners’ knowledge, and variable emphasis can thus be given to questions or topics according to the learners’ needs. Even if no measurable increase in knowledge specifically attributable to ARS is quantified, it may still be a useful tool to rapidly assess learners and help instructors provide learner-centered education [6].

Similar to what we found in our surveys, it is reported in the literature that the use of ARS facilitates student engagement and learning [9], as students are prompted to answer questions posted at variable times throughout the lectures. Three studies demonstrated a statistically significant difference in knowledge and assessment scores that favored ARS versus non-ARS teaching [9]. In addition, other studies suggest ARSs serve to improve learners’ attention and enthusiasm and promote confidence for conference attendance [17-19]. Additionally, in settings such as residency training programs where sleep deprivation and subsequent difficulties with attention and clear thinking are common, the ability of ARS to enhance learner interactivity is potentially very beneficial [9]. With SCORE MCQs, participation in a relaxed environment combined with ARS use is useful to enhance engagement and interactivity.

We noted an interesting difference between faculty and learners in their assessment of ARS. Although both groups preferred it and felt that it was better than traditional lectures (demonstrated in the survey results and elucidated within the focus group discussion), only approximately half of the trainees felt that it was an efficient use of learning time, and a similar number felt that their experience with ARS encouraged them to further use technology in education. By comparison, all the faculty members uniformly felt that using ARS was efficient and induced a desire for further use of technology. We hypothesize that these differences in perspective, based on what both agreed was a positive ARS experience, reflect generational differences, with the younger trainees already being well versed in technology and its educational utility in multiple settings.

In the FGD, engagement and thinking stimulation were among the most often cited positive benefits. There was no significant perceived negative impact in the study group. Our study has contributed to the body of research on the efficacious use of ARS in a specific setting with various types of learners. However, the study has only covered a small subset of potential applications for ARS use in surgical education. Future studies may investigate larger resident and medical student populations to explore the potential socio-demographic impact on ARS preferences and possible variations in efficacy for different surgical education curricula and educational settings. ARS platforms have evolved, and there are now more options employing evolving technology, such as digital ARS optimized for use on personal smartphones rather than hand-held proprietary clickers. These options are more cost-effective and more portable.

Several potential challenges may impede the wide-scale implementation and use of ARS in medical education. The introduction of new technology may lead to difficulties with the adoption and troubleshooting of technical issues, with the burden typically falling on the individual instructor. The cost of the ARS could be a factor for some institutions; however, free web-based software is becoming increasingly available. Differences in software and proprietary idiosyncrasies may lead to confusion and frustration among learners as they go into different learning environments. Institutional traditions and individual teaching practices may make change difficult. Inevitably, the use of ARS will require additional time and effort to fully incorporate and embed into each educational event. This may lead to an increased workload for instructors, who may already feel overstretched. Occasionally, as we have seen, some learners may consider the ARS a waste of "real education" time.

The study has several limitations. Some of these limitations are inherent to the methods chosen and the limited sample size. In a typical flipside of surveys, our respondents were self-selected and may not adequately represent the whole body of faculty, residents, and students. Focus group discussions could be biased as the opinions of certain group members could be influenced by more dominant contributors. Furthermore, the study does not measure all aspects of the educational process and potential outcomes of introducing a new tool. The study focuses on the interactive component of the educational process as perceived by users. Even though perception plays an essential role in stimulating active learning, it is a subjective measure that is difficult to quantify, compare, or follow. Measuring other educational outcomes, such as improved clinical performance, higher grades, or improved retention of knowledge, requires a separate and larger study.

## Conclusions

The use of ARS in teaching *This Week In SCORE* sessions, it is advantageous. It is a practical and efficient use of educational technology that has been well received by a majority of faculty, as well as residents and medical students. The most frequently mentioned benefits were ease of use and facilitating engagement and interactivity. ARS, in its various forms or versions within educational technology, requires further research to explore more efficient learning application strategies. Among the potentially promising areas, gamification and connectivism in learning stand out as two fields where additional studies could prove fruitful.

## Appendices

Appendix A: Faculty Survey Form

1- Using ARS and SCORE questions needed an acceptable amount of preparation time and effort.

Strongly agree. Agree. Neutral. Disagree. Strongly disagree.

2- Using ARS and SCORE questions helped learners’ engagement and participation in the learner-centered educational activity.

Strongly agree. Agree. Neutral. Disagree. Strongly disagree.

3- Using ARS and SCORE questions helped knowing learners’ levels and facilitated building on their knowledge.

Strongly agree. Agree. Neutral. Disagree. Strongly disagree.

4- Using ARS and SCORE questions facilitated learning relevant to the daily residents’ clinical activities.

Strongly agree. Agree. Neutral. Disagree. Strongly disagree.

5- Using ARS and SCORE questions was an efficient use of learning time.

Strongly agree. Agree. Neutral. Disagree. Strongly disagree.

6- I enjoyed using ARS and SCORE questions. It is educationally better than traditional lectures.

Strongly agree. Agree. Neutral. Disagree. Strongly disagree.

7- Using ARS and SCORE questions was an appropriate way to teach the topic of the week.

Strongly agree. Agree. Neutral. Disagree. Strongly disagree.

8- Using ARS and SCORE questions introduced and encouraged using technology in training.

Strongly agree. Agree. Neutral. Disagree. Strongly disagree.

9- Using ARS and SCORE questions facilitated deeper learning, focusing more on concepts and principles than factual information.

Strongly agree. Agree. Neutral. Disagree. Strongly disagree.

10- Overall, using ARS and SCORE questions was a good quality educational activity.

Strongly agree. Agree. Neutral. Disagree. Strongly disagree.

Appendix B: Residents and Students Survey Form

1 - Using ARS and SCORE questions was easy and practical.

Strongly agree. Agree. Neutral. Disagree. Strongly disagree.

2 - Using ARS and SCORE questions was motivational and engaging

Strongly agree. Agree. Neutral. Disagree. Strongly disagree.

3 - Using ARS and SCORE questions was stimulating to study and learn the topic.

Strongly agree. Agree. Neutral. Disagree. Strongly disagree.

4 - Using ARS and SCORE questions helped me better understand and apply knowledge.

Strongly agree. Agree. Neutral. Disagree. Strongly disagree.

5 - Using ARS and SCORE questions encouraged me to use technology in my learning.

Strongly agree. Agree. Neutral. Disagree. Strongly disagree.

6 - Using ARS and SCORE questions encouraged participants to discuss topics with colleagues and faculty.

Strongly agree. Agree. Neutral. Disagree. Strongly disagree.

7 - Using ARS and SCORE questions helped in improving the quality of learning and focusing on principles, concepts, approaches, surgical thinking, and decision-making.

Strongly agree. Agree. Neutral. Disagree. Strongly disagree.

8 - Using ARS and SCORE questions was an efficient use of learning time.

Strongly agree. Agree. Neutral. Disagree. Strongly disagree.

9 - Using ARS and SCORE questions was inspiring to learn deeper and study the topic further.

Strongly agree. Agree. Neutral. Disagree. Strongly disagree.

10 - I prefer using ARS and SCORE questions compared to routine, traditional lectures or discussions.

Strongly agree. Agree. Neutral. Disagree. Strongly disagree.

## References

[1] Alaagib NA, Musa OA, Saeed AM (2019). Comparison of the effectiveness of lectures based on problems and traditional lectures in physiology teaching in Sudan. BMC Med Educ.

[2] Ikpeze CH (2015). Teaching Across Cultures: Building Pedagogical Relationships in Diverse Contexts. Sense Publishers.

[3] Chilwant KS (2012). Comparison of two teaching methods, structured interactive lectures and conventional lectures. Biomedical Research.

[4] Padgett J, Cristancho S, Lingard L, Cherry R, Haji F (2019). Engagement: what is it good for? The role of learner engagement in healthcare simulation contexts. Adv Health Sci Educ Theory Pract.

[5] Caldwell JE (2007). Clickers in the large classroom: current research and best-practice tips. CBE Life Sci Educ.

[6] Chung H, Kallay T, Anas N (2018). Using an audience response system smartphone app to improve resident education in the pediatric intensive care unit. J Med Educ Curric Dev.

[7] Shah KH, Jordan J, Jahnes K (2017). Audience response system facilitates prediction of scores on in-training examination. West J Emerg Med.

[8] Leeds IL, DiBrito SR, Jones CD, Higgins RS, Haut ER (2018). Using audience response systems for real-time learning assessments during surgical morbidity and mortality conference. J Surg Educ.

[9] Nelson C, Hartling L, Campbell S, Oswald AE (2012). The effects of audience response systems on learning outcomes in health professions education. A BEME systematic review: BEME Guide No. 21. Med Teach.

[10] Gubrium JF, Holstein JA (2001). Handbook of Interview Research. Handbook of Interview Research.

[11] Creswell J. Developing Mixed Methods Research with Dr. John W. Creswell (2020). YouTube: Developing mixed methods research with Dr. John W. Creswell. https://www.youtube.com/watch?v=PSVsD9fAx38.

[12] (2020). DI: Research tools: focus group discussion. https://www.odi.org/publications/5695-research-tools-focus-group-discussion.

[13] Krippendorff K (2004). Content Analysis: An Introduction to Its Methodology. Thousand Oaks, CA: Sage.

[14] Weber RP (1990). Basic Content Analysis.

[15] Creswell J (2020). YouTube: What is mixed methods research. https://www.youtube.com/watch?v=1OaNiTlpyX8   .

[16] Nayak L, Erinjeri JP (2008). Audience response systems in medical student education benefit learners and presenters. Acad Radiol.

[17] Miller RG, Ashar BH, Getz KJ (2003). Evaluation of an audience response system for the continuing education of health professionals. J Contin Educ Health Prof.

[18] Mains TE, Cofrancesco J Jr, Milner SM, Shah NG, Goldberg H (2015). Do questions help? The impact of audience response systems on medical student learning: a randomised controlled trial. Postgrad Med J.

[19] Doucet M, Vrins A, Harvey D (2009). Effect of using an audience response system on learning environment, motivation and long-term retention, during case-discussions in a large group of undergraduate veterinary clinical pharmacology students. Med Teach.

